# Adlay Testa (*Coix lachryma-jobi* L. var. *Ma-yuen* Stapf.) Ethanolic Extract and Its Active Components Exert Anti-Proliferative Effects on Endometrial Cancer Cells via Cell Cycle Arrest

**DOI:** 10.3390/molecules26071966

**Published:** 2021-03-31

**Authors:** Yun-Ju Huang, Chih-Chao Chang, Yun-Ya Wang, Wen-Chang Chiang, Yin-Hwa Shih, Tzong-Ming Shieh, Kai-Lee Wang, Mohamed Ali, Shih-Min Hsia

**Affiliations:** 1 School of Nutrition and Health Sciences, College of Nutrition, Taipei Medical University, Taipei 110301, Taiwan; d04641004@ntu.edu.tw (Y.-J.H.); changhcym@gmail.com (C.-C.C.); 2 College of Bioresources and Agriculture, National Taiwan University, Taipei 10617, Taiwan; r99641036@ntu.edu.tw (Y.-Y.W.); chiang@ntu.edu.tw (W.-C.C.); 3 Department of Healthcare Administration, Asia University, Taichung 41354, Taiwan; evashih@asia.edu.tw; 4 School of Dentistry, College of Dentistry, China Medical University, Taichung 404333, Taiwan; tmshieh@mail.cmu.edu.tw; 5 Department of Dental Hygiene, College of Health Care, China Medical University, Taichung 404333, Taiwan; 6 Department of Nursing, Ching Kuo Institute of Management and Health, Keelung City 203301, Taiwan; kellywang@tmu.edu.tw; 7 Clinical Pharmacy Department, Faculty of Pharmacy, Ain Shams University, 11566 Cairo, Egypt; mohamed.aboouf@pharma.asu.edu.eg; 8 Nutrition Research Center, Taipei Medical University Hospital, Taipei 110301, Taiwan; 9 School of Food and Safety, Taipei Medical University, Taipei 110301, Taiwan; 10 Graduate Institute of Metabolism and Obesity Sciences, College of Nutrition, Taipei Medical University, Taipei 110301, Taiwan

**Keywords:** Adlay, polyphenol, flavonoids, phytosterols, inhibitory effects

## Abstract

Endometrial cancer is the most common malignant tumors of gynecologic neoplasms in Western society. In recent years, the incidence of endometrial cancer has increased, and it has become the third most common female gynecological cancer (after ovarian and cervical cancer) in Taiwan. Adlay (*Coix lachryma-jobi* L. var. *Ma-yuen* Stapf.) has been demonstrated to have bioactive polyphenols, flavonoids, phytosterols, and essential nutrients for health benefits, including anticancer effects in humans. However, little is known about the effect of adlay seeds on endometrial cancer. Our study aimed to investigate the potential growth inhibitory effects of several adlay seed fractions, including ethyl acetate (ATE-EA) and its bioactive constituents, separately on endometrial cancer cells—HEC-1A (phosphatase and tensin homolog-positive) and RL95-2 (phosphatase and tensin homolog-negative)—and identify related active ingredients. In addition, the potential active fractions and the phytochemical compounds were elucidated. The results demonstrate superior activity of ATE-EA with significant in vitro cell proliferation inhibitory capacity, particularly its C.D.E.F-subfraction. Moreover, HPLC- and GC/FID-based quantification of ATE-EA subfractions showed that phenolic compounds (caffeic acid, protocatechuic acid, and p-hydroxybenzaldehyde), flavonoids, steroids, and fatty acid compounds exert anti-proliferative effects in the cell model. Finally, it was shown that cell growth and cell cycle arrest most significantly occurred in the in G1 or G2/M phase under ATE-EA treatment. Collectively, our results demonstrate an antiproliferative effect of ATE-EA on endometrial cancer cells that suggest a positive health outcome for women from consumption of these compounds.

## 1. Introduction

Endometrial cancer is the most common malignant tumor in women in developed countries, and the fifth most common cancer in women worldwide [1]. The mortality rate of endometrial cancer has at least doubled over the past 20 years and increased by 8% since 2008 [2]. The incidence of endometrial cancer has increased by 37.52 per 100,000 women aged between 55 and 59 years annually in Taiwan [3]. In recent decades, the high incidence of obesity and metabolic syndrome-related diseases are considered to be among the potential risk factors for endometrial cancer in Taiwan [4]. Notably, the incidence rate has been increasingly reported in younger women, with up to 14% of newly diagnosed cases occurring in premenopausal women, and 5% of patients are younger than 40 years [5,6]. Standard treatment of endometrial cancer consists of surgery, chemoradiation therapy, chemotherapy, and molecular targeted therapies, with high risk of tumor recurrence. The ideal management of endometrial cancer would offer optimal survival with the lowest risk of recurrence and adverse effects [7]. 

Coix (*Coix lachryma-jobi* L. var. *Ma-yuen* Stapf.), also known as adlay, has been used in traditional Chinese medicine and food for a long time. In recent years, more research interest is being directed towards adlay biological activities, including its anti-cancer [8], anti-inflammation [9], and anti-allergic effects [10] as well as others [11]. For example, adlay seed extract showed anti-diabetic activity of its polysaccharides [12], antioxidant activity of its oil [13], and antiallergic properties of its phenolic compounds [10]. The seeds produce cytotoxic lactams in the bran [8] and antioxidative lignans in the hulls [14]. The major fatty acids of the seeds are oleic (46.3%) and linoleic acid (37.4%) [15]. Studies have indicated that higher intake of monounsaturated fatty acids is negatively correlated with the risk of endometrial cancer [16]. The antioxidant and anti-cancer effects of phenolic compounds encourage the design of novel synthetic drugs [17]. In women’s health, two phytosterols, stigmasterol and β-sitosterol, from the ethyl acetate fraction of adlay hull reduced uterine myometrial hyperplasia induced in a mouse model [18]. Additionally, four major flavonoids constituents from adlay hull ethanolic extract exhibited inhibition on polycystic ovary syndrome (PCOS) [19].

At the cellular level of cancer treatment, it has been suggested that adlay is likely to influence proliferation in various cell lines, including A549, MCF-7, and HT-29 [20,21]. Adlay extracts also showed high anti-proliferative efficiency against both rat uterine leiomyoma cells (ELT3) and primary human uterine leiomyoma cells (HuLM) [14] and human histolytic lymphoma U937 monocytic cells [22]. Nonetheless, little information is available in the literature about the chemical compositions of adlay testa (ATE) ethanolic extracts as well as their efficacy in limiting endometrial cancer cell proliferation. In the present study, we investigated the growth inhibitory effects of the ATE extract on endometrial cancer cell lines, including HEC-1A (phosphatase and tensin homolog-positive) and RL95-2 (phosphatase and tensin homolog-negative), as well as fractionation testing to elucidate the active fractions and effective components. 

## 2. Results

### 2.1. Effects of Different Parts of the Adlay on Endometrial Cancer Cell Viability

**Figure 1:**
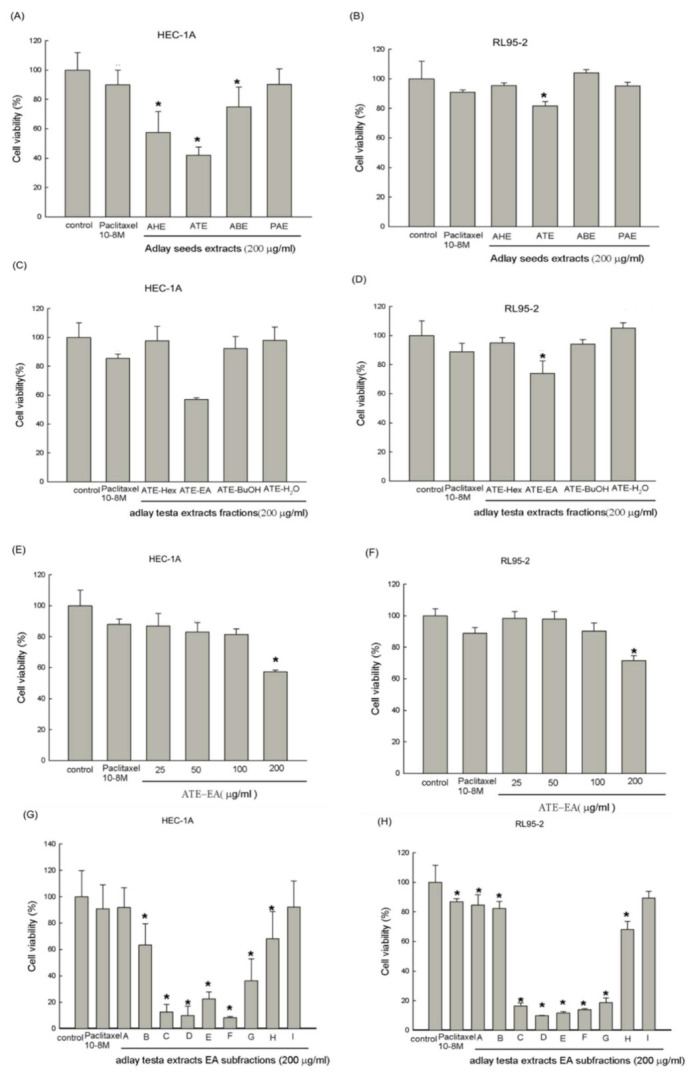
Effects of adlay seed ethanolic extracts on the viability of HEC-1 and RL95-2 cells. The effect of ethanolic extracts from different parts of the adlay seed on the viability of HEC-1 (**A**) and RL95-2 (**B**) cells. The effect of different fractions of adlay testa ethanolic extract (ATE) on the growth of HEC-1A (**C**) and RL95-2 (**D**) cells. HEC-1A cells and RL95-2 cellswere treated with different concentrations (**E**,**F**) and subfractions (**G**,**H**) of the ethyl acetate fraction of the adlay testa ethanolic extract (ATE-EA) for 48 h, respectively. Data are expressed as means ± SD (*n* = 3). * *p* < 0.05, compared with the control group at the basal level. AHE, ethanolic extracts of adlay hull; ATE, ethanolic extracts of adlay testa; ABE, ethanolic extracts of adlay bran; PAE, ethanolic extracts of polished adlay; ATE-Hex, n-hexane fraction of adlay testa ethanolic extract; ATE-BuOH, 1-butanol fraction of adlay testa ethanolic extract; ATE-H_2_O, H_2_O fraction of adlay testa ethanolic extract.

Cell viability was assessed using the 3-(4,5-Dimethylthiazol-2-yl)-2,5-diphenyltetrazolium bromide (MTT) assay to verify the effect of ethanol extracts of four parts of the adlay seed in human endometrial cancer cells (HEC-1A and RL95-2). Ethanol extracts of the hull, testa, bran, and polish adlay of the seed are referred to as AHE, ATE, ABE, and PAE, respectively. The cells were treated with AHE, ATE, ABE, and PAE (200 μg/mL) for 48 h. As shown in Figure 1A,B, ATE treatment significantly reduced the viability of HEC-1A and RL95-2 cells at 48 h. PAE only slightly reduced HEC-1A cell viability at 48 h. AHE, ABE, and PAE showed no effect on RL95-2 cell viability at 48 h. These results suggest that ATE exerted potential anti-cancer effects compared to the other components. Therefore, ATE was subjected to further investigation. This study used paclitaxel, a standard initial therapy for advanced endometrial cancer, as a positive control.

### 2.2. Effects of Different Fractions and Subfractions of Adlay Testa on Endometrial Cancer Cell Viability

The potential anticancer effect of different ATE fractions on the proliferation of human endometrial cancer cells (HEC-1A, and RL95-2) were screened using MTT. The cells were treated with ATE-Hex, ATE-EA, ATE-Bu, and ATE-H_2_O at 200 μg/mL for 48 h. The results show that the ATE-EA fraction significantly reduced the viability of RL95-2 cells (Figure 1D), but. in HEC-1A (Figure 1C), a statistical trend was found at 48 h. ATE-Hex and ATE-Bu fractions only slightly reduced HEC-1A (Figure 1C) and RL95-2 cell (Figure 1D) viability at 48 h. Finally, the cell viability assay showed no significant difference between the untreated control and ATE-H_2_O groups at 48 h on both cell lines. Collectively, these results indicate that major anti-cancer components possibly exist in the lower-polarity fraction of ATE, especially ATE-EA. Therefore, the ATE-EA fraction was selected for further investigation.

The MTT cell proliferation assay was then performed to further assess the effects of different concentrations of ATE-EA on human endometrial cancer cell (HEC-1A and RL95-2) proliferation. We treated cells with ATE-EA ranging from 25 to 200 μg/mL for 48 h. As shown in Figure 1E,F, ATE-EA significantly inhibited the viability of HEC-1A cells (Figure 1E) and RL95-2 cells (Figure 1F) at 200 μg/mL at 48 h, compared to the untreated control. These data suggest a selective targeting of human endometrial cancer cells by ATE-EA. Later, chromatographic subfractions of the ATE ethyl acetate fraction (ATE-EA), nine samples in total, were screened for cytotoxic activity on human endometrial cancer cells (HEC-1A and RL95-2) using the MTT assay, and the results are presented in Figure 1G, H. ATE-EA-A–H chromatographic subfractions showed potent growth inhibition effects on both HEC-1A (Figure 1G) and RL95-2 (Figure 1H) cells at 48 h. These data suggest that these chromatographic subfractions exert anti-proliferation activity against human endometrial cancer cells. 

### 2.3. Phenolic Compounds in ATE-EA Inhibited Endometrial Cancer Cells Growth 

**Figure 2:**
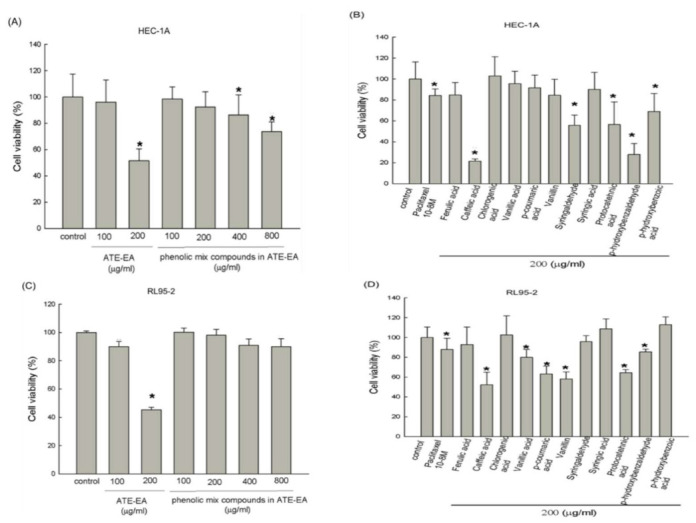
Effects of phenolic compounds from ATE-EA on the viability of HEC-1A and RL95-2 cells. HEC-1A cells were treated with serial concentrations of phenolic compound mixture (**A**) and its individual constituents at concentrations of 200 μg/mL (**B**) of ATE-EA for 48 h. RL95-2 cells were treated with serial concentrations of phenolic compounds (**C**) and their constituents at a concentration of 200 μg/mL (**D**). Cell viability was detected using an MTT assay. Data are expressed as means ± SD (*n* = 3). * *p* < 0.05, compared with the control group at the basal level. ATE-EA, ethyl acetate fraction of the adlay extract.

The growth inhibitory effect of 11 phenolic compounds (identified in ATE-EA as follows: protocatechnic acid, *p*-hydroxybenzoic acid, chlorogenic acid, vanillic acid, *p*-hydroxybenzaldehyde, syringic acid, vanillin, syringaldehyde, caffeic acid, *p*-coumaric acid, and ferulic acid) were explored in HEC-1A and RL95-2 cells using the MTT assay. Phenolic compounds in ATE-EA were identified by HPLC analysis based on the reference [23]. The results, as shown in Figure 2A,C, display growth inhibitory effects of the phenolic compounds mixture at serial concentrations (100, 200, 400, and 800 μg/mL) on both HEC-1A and RL95-2 cells at 48 h, compared to ATE-EA (100 and 200 μg/mL). This result suggests that the ATE-EA at the 200 ug/mL dose has a better inhibitory effect than that phenolic mixtures. Moreover, the individual growth inhibitory effect of these 11 phenolic compounds were explored (200 μg/mL each) compared to the untreated control to identify their relative anti-cancer effects on both HEC-1A and RL95-2 cells using the MTT assay. The results suggest that protocatechnic acid, caffeic acid, and *p*-hydroxybenzaldehyde inhibited the proliferation of HEC-1A and RL95-2 cells (Figure 2B, D). HEC-1A and RL95-2 cells were more sensitive to ATE-EA than the mixed phenolic compounds at same concentrations (200 μg/mL).

### 2.4. Flavonoid Compounds Inhibited Endometrial Cancer Cell Growth

**Figure 3:**
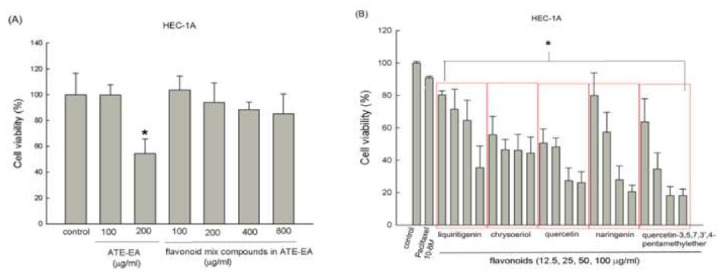

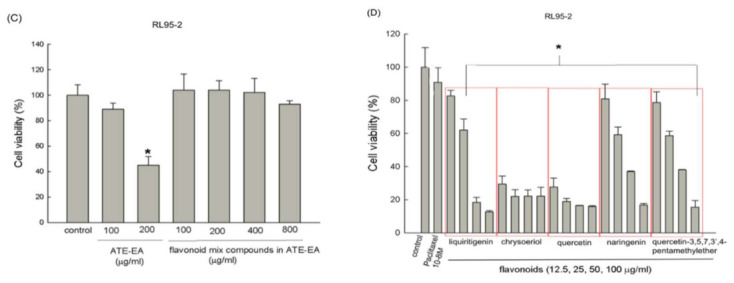
Effects of flavonoid compounds from ATE-EA on the viability of HEC-1A and RL95-2 cells. HEC-1A cells were treated with serial concentrations of flavonoid compounds (**A**) and the constituents (**B**) of ATE-EA for 48 h. RL95-2 cells were treated with serial concentrations of flavonoid compounds (**C**) and the constituents (**D**) of ATE-EA for 48 h. Data are expressed as means ± SD (*n* = 3). * *p* < 0.05, compared with the control group at the basal level. ATE-EA, ethyl acetate fraction of the adlay testa ethanolic extract.

We evaluated the potential anti-cancer effects of five flavonoid compounds (liquiritigenin, chrysoeriol, quercetin, naringenin, and quercetin-3,5,7,3’,4-pentamethylether) individually as well as combined at different four concentrations in HEC-1A and RL95-2 cells (Figure 3A–D). The flavonoid contents were analyzed by HPLC based on the reference [24]. The results confirm the previous findings that ATE-EA reduced cell growth when the dosage was 200 μg/mL. The mixed flavonoid compounds suppressed cell growth in vitro and showed only a decreasing trend. Our results indicate that liquiritigenin, chrysoeriol, quercetin, naringenin, and quercetin-3,5,7,3’,4-pentamethylether produced anti-proliferative effects at different doses (12.5, 25, 50, and 100 μg/mL) in the HEC-1A cell line. The red boxes represent the individual flavonoid compounds with series concentrations.

### 2.5. Steroids Inhibited Endometrial Cancer Cells Growth 

**Figure 4:**
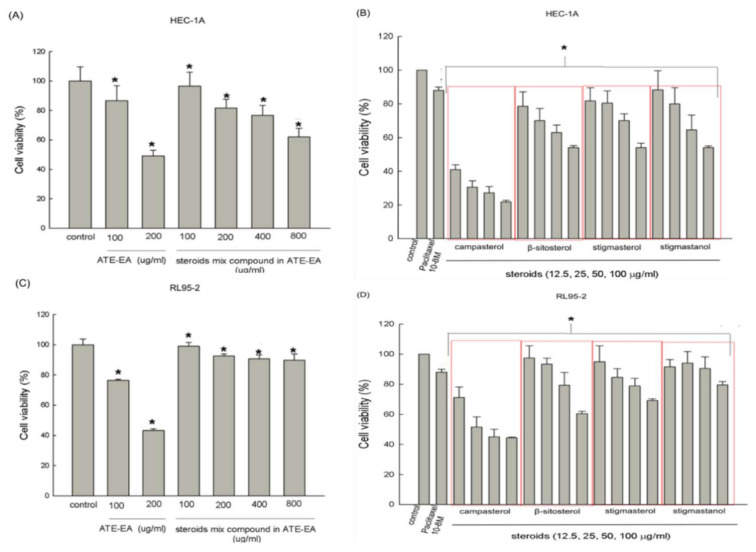
Effects of steroid compounds from ATE-EA on the viability of HEC-1A and RL95-2 cells. HEC-1A cells were treated with serial concentrations of steroid compounds (**A**) and the constituents (**B**) of ATE-EA for 48 h. RL95-2 cells were treated with serial concentrations of steroid compounds (**C**) and the constituents (**D**) of ATE-EA for 48 h. Cell viability was detected using an MTT assay. Data are expressed as means ± SD (*n* = 3). * *p* < 0.05, *p* < 0.01, compared with the control group at the basal level. ATE-EA, ethyl acetate fraction of the adlay testa ethanolic extract.

The potential growth inhibitory effect of the mixture of steroid compounds as well as four steroids in ATE-EA, including campesterol, β-sitosterol, stigmasterol, and stigmastanol, were explored on HEC-1A and RL95-2 cells using the MTT assay (Figure 4A–D) and compared to ATE-EA at the doses of 100 and 200 μg/mL over 48 h. The results suggest that steroids partly governed the inhibitory ability of ATE-EA. These results are consistent with ATE-EA at the optimal dose of 200 μg/mL. The results demonstrate that HEC-1A and RL95-2 cells were more sensitive to ATE-EA than the four mixed steroids. The red boxes represent the individual steroid compounds with the serial concentrations.

### 2.6. Fatty Acid Compounds Inhibited HEC-1A and RL95-2 Cells Growth

**Figure 5:**
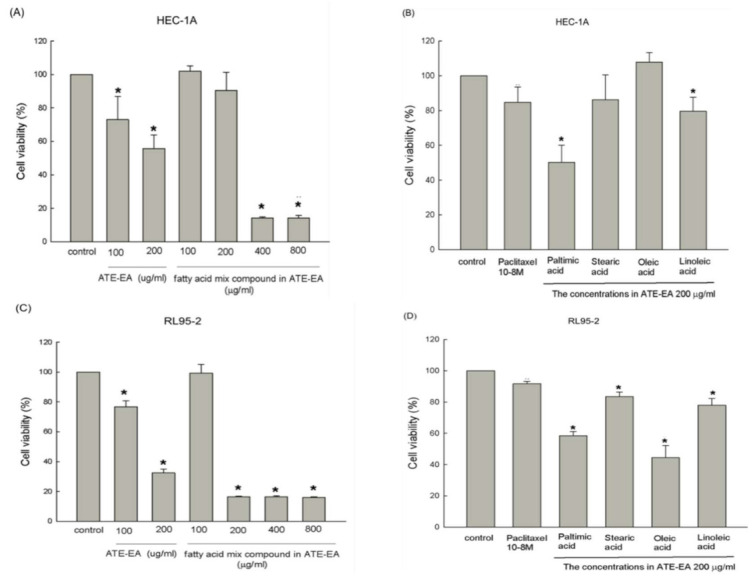
Effects of fatty acid compounds from ATE-EA on the viability of HEC-1A and RL95-2 cells. HEC-1A cells were treated with serial concentrations of fatty acid compounds (**A**) and its constituents at a concentration of 200 μg/mL (**B**) of ATE-EA for 2 days. RL95-2 cells were treated with serial concentrations of fatty acid compounds (**C**) and its constituents at a concentration of 200 μg/mL (**D**). Data are expressed as means ± SD (*n* = 3). * *p* < 0.05, compared with the control group at the basal level. ATE-EA, ethyl acetate fraction of the adlay testa ethanolic extract.

We evaluated the proliferation suppressive efficiency of the fatty acid mixture as well as four individual fatty acids, including palmitic acid, stearic acid, oleic acid, and linoleic acid, of ATE-EA on both HEC-1A and RL95-2 cells using the MTT assay (Figure 5A–D). The results show that treatment with ATE-EA (200 μg/mL) and the mix of fatty acid compounds at both 400 and 800 ug/mL for 48 h significantly reduced HEC-1A and RL95-2 cell growth. Both palmitic acid and linoleic acid at the optimal dose showed a considerably strong inhibitory effect on HEA-1A and RL95-2 cell lines. A similar trend was observed for ATE-EA, which compared better to the mixed compounds in these cell assays. 

### 2.7. Four Mixture Compounds Inhibited HEC-1A and RL95-2 Cells Growth

**Figure 6:**
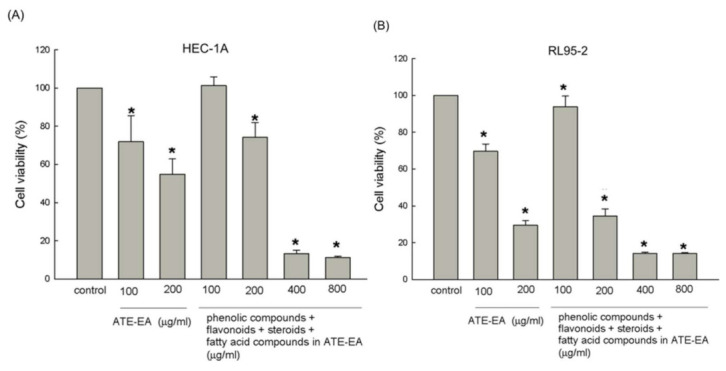
Effects of the mixture of four compounds from the ATE-EA on the viability of HEC-1A and RL95-2 cells. HEC-1A (**A**) and RL95-2 (**B**) cells were treated with serial concentrations of ATE-EA and four mixed compounds at a concentration of 200–800 μg/mL for 48 h. Data are expressed as means ± SD (*n* = 3). * *p* < 0.05, compared with the control group at the basal level. ATE-EA, ethyl acetate fraction of the adlay testa ethanolic extract.

To better understand the relationship between the inhibitory effects and the mixtures of phenolics, flavonoids, steroids, and fatty acids in the ATE-EA, the above four mixtures of compounds were compared to the ATE-EA. The results suggest that the inhibitory effect of ATE-EA on cell growth was concentration-dependent. In addition, the strongest cell growth inhibition was observed and increased with the dosage of the four mixed compounds at concentrations of 400 and 800 μg/mL in both HEC-1A and RL95-2 cell lines (Figure 6A,B). Therefore, the involved phenolic components—flavonoids, steroids, and fatty acids of ATE-EA—contributed to the anti-inhibitory activities on endometrial cancer cells.

### 2.8. ATE-EA Induced G2/M and Sub-G1 Phase Arrest in Endometrial Cancer Cells

**Figure 7:**
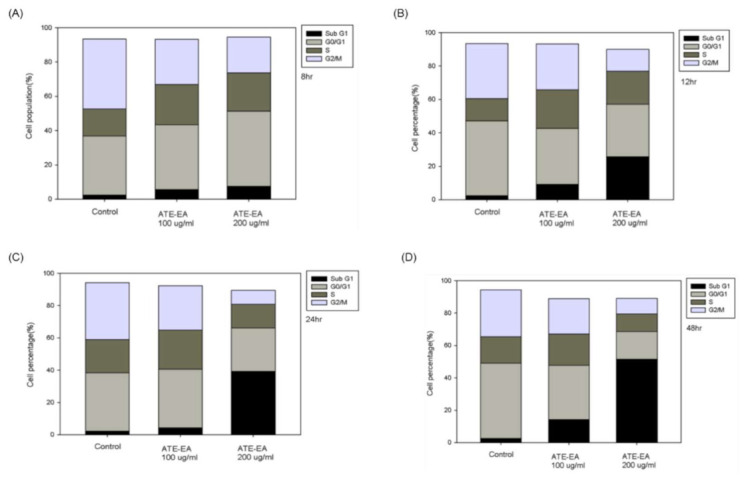

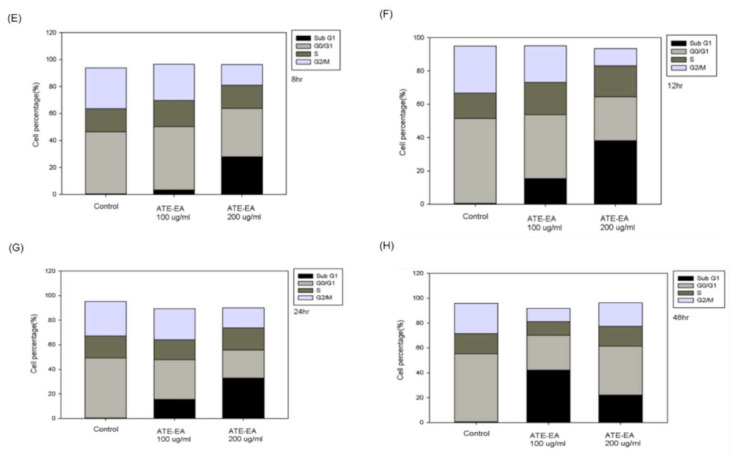
The effects of ATE-EA on the cell cycle distribution. HEC-1A cells were treated ATE-EA at 100 and 200 μg/mL for: (**A**) 8 h; (**B**) 12 h; (**C**) 24 h; and (**D**) 48 h. RL95-2 cells were treated ATE-EA at 100 and 200 μg/mL for: (**E**) 8 h; (**F**) 12 h; (**G**) 24 h; and (**H**) 48 h. Analysis of the cell cycle was done by flow cytometry. Representative histograms of cell cycle progression. 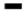 Sub-G1; 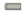 G0/G1; 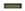 S; 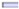 G2/M. ATE-EA, ethyl acetate fraction of the adlay testa ethanolic extract.

To identify whether the cytotoxic effect of ATE-EA is associated with induction of cell cycle arrest, HEC-1A and RL95-2 cells were treated with ATE-EA at different concentrations (100 and 200 μg/mL) for 8, 12, 24, and 48 h and changes in cell cycle distribution were measured using flow cytometry (Figure 7A–H). ATE-EA (100 μg/mL) treatment for 8, 12, 24, and 48 h increased the percentage of HEC-1A and RL95-2 cells in the G0/G1 phase. Doubling the concentration of ATE-EA to 200 μg/mL increased the sub-G1 phase at 12, 24, and 48 h. Collectively, ATE-EA showed dose-dependent cell cycle arrest of HEC-1A and RL95-2 cells at the sub G1 checkpoint and G2/M checkpoint. 

### 2.9. Identification of the Main Phenolic and Flavonoid Compounds in ATE-EA by HPLC

The different phenolic compounds presented in ATE-EA were characterized using high performance liquid chromatography (HPLC) analysis. Figures S1 and S2 show the chromatograms of the phenolic compound analysis and the reference standards. Briefly, identifying the major phenolic compounds in ATE-EA was done by comparing the chromatograms of analyzed samples to those of the standards (protocatechnic acid, *p*-hydroxybenzoic acid, chlorogenic acid, vanillic acid, *p*-hydroxybenzaldehyde, syringic acid, vanillin, syringaldehyde, caffeic acid, *p*-coumaric acid, and ferulic acid). This analysis allowed identification of the retention times of the phenolic compounds presented in ATE-EA as well as the retention times relative to the standard via HPLC analysis (see Figures S1 and S2 and Table S2 in Supplementary Materials). The major phenolic and flavonoid compounds in ATE-EA are shown in Table 1 and Table 2 (cf. Table S3), respectively. In addition, five major flavonoid compounds, namely chrysoeriol, liquiritigenin, naringenin, quercetin, and quercetin-3,5,7,3’,4-pentamethylether, were identified in ATE-EA by the curves of the five major flavonoid standards.

### 2.10. Identification of the Phytosterols in ATE-EA by GC/FID

The different phytosterols in ATE-EA were identified using gas chromatography (GC)/flame ionization detection (FID). This chromatographic method proved efficient at detecting and separating steroids as it can be applied to different types of detectors with no loss of analytical sensitivity. Figure S3 shows the analysis of ATE-EA and the relevant reference standards. Individual phytosterols in ATE-EA were identified by comparing their retention time with those of the available standards (β-sitosterol, stigmasterol, stigmastanol, and campesterol) and the retention time relative to the internal standard cholesterol via GC/FID. The major phytosterols in ATE-EA are shown in Table 3. The phytosterols were eluted at time points ranging from 20.20 to 22.15 min. The internal standard used in quantifying the phytosterols was eluted in 18.36 min.

## 3. Discussion

This study used a cell model to demonstrate that the ethyl acetate fraction of the adlay extract (ATE-EA) was able to inhibit proliferation of endometrial cancer cells in vitro. Using a solvent extraction technique, the ethyl acetate fraction was extracted from ATE (ATE-EA); ATE-EA was further divided into nine subfractions A–I using gel filtration. We found that Subfractions C–F (ATE-EA-C–F) at a lower concentration (200 μg/mL) suppressed HEC-1-A and RL95-2 cell growth. Analysis of ATE-EA using HPLC and GC/FID identified phenolic compounds, flavonoids, steroids, and fatty acid compounds which exhibited the identified inhibitory effects. Moreover, our results indicate that the anti-proliferative efficiency of ATE-EA on endometrial cancer cells was higher than the above active compounds alone, thus offering a potential therapeutic option for women with endometrial cancer.

Previous studies showed that methanol extracts of adlay bran exerted an anti-proliferative effect on human lung cancer cells A549, human colorectal carcinoma cells HT-29 [8], and breast cancer [20,21]. Another study indicated that the methanolic extract of adlay seed may inhibit the proliferation of A549 lung cancer cells through downregulating cyclin A expression and induction of apoptotic cell death [25]. We previously showed that the ethyl acetate fraction of adlay hull (AHE-EA) inhibited the growth of both rat uterine leiomyoma cells (ELT3) and primary human uterine leiomyoma cells [18]. Recently, we demonstrated that the hexane fraction of the adlay testa ethanolic extracts (ATE-Hex) can inhibit human uterine sarcoma cancer cells via apoptosis induction and enhancement of the chemosensitivity of the multidrug-resistant human uterine sarcoma cancer cell MES-SA/Dx5 to doxorubicin [26]. However, there is limited information on adlay testa antiproliferative effects on endometrial cancer cells. This study is the first to investigate the potential effects of adlay extracts using an endometrial cancer cell model. Interestingly, we found that ATE-EA is associated with cell cycle arrest in G1 or G2/M phase.

Phenolic compounds are considered among the major bioactive compounds in plant materials [27]. The structural hydroxyl groups within the phenolic compounds account for the capability of these compounds in scavenging reactive oxygen species, such as superoxide radicals, singlet oxygen, hydroxyl radicals, nitric oxide, nitrogen dioxide, and peroxynitrite, thus pertaining to their antioxidant and potential anti-cancer capacities [28]. Several phenolic antioxidants were isolated from adlay seeds that had stable bioactivity during processing [29]. Examples of phenolic compounds with known antioxidants and anticancer effects are caffeic acid and other polyphenols such as ferulic acid, gallic acid, *p*-coumaric acid, and vanillic acid [30]. In the present study, ATE-EA was found to contain phenolic compounds: protocatechnic acid, *p*-hydroxybenzoic acid, chlorogenic acid, vanillic acid, *p*-hydroxybenzaldehyde, syringic acid, vanillin, syringaldehyde, caffeic acid, *p*-coumaric acid, and ferulic acid. Cell viability analysis showed that human endometrial cancer cells (HEC-1-A and RL95-2) were affected by ATE-EA at 200 µg/mL. Moreover, the same cells were also affected by the ATE-EA extracts and the mixed phenolic compounds.

Flavonoids are a large group of plant polyphenol secondary metabolites divided into six classes based on their structure. They exist widely in the leaves, seeds, bark, and flowers of plants [31]. Flavonoids include flavanones, flavones, flavonols, isoflavonoids, anthocyanins, and flavans [32]. Flavonoids exhibit strong antioxidant capacities by scavenging oxygen free radicals, promoting anti-oxidase enzymes, or inhibiting oxidative enzymes [33]. Three flavonoid compounds (rutin, quercetin, and kaempferol) from defatted adlay seed were examined. Among these three flavonoid compounds, quercetin exhibited the highest peroxyl radical scavenging capacity values and cellular antioxidant activity values [34]. Notably, Chen et al. reported 10 flavonoid compounds with anti-inflammatory effects isolated from adlay bran [24]. Meanwhile, a previous study revealed that quercetin, nobiletin, eriodictyol, sitosterol, stigmasterol, and stigmastanol significantly decreased the viability of ELT3 and primary leiomyoma cells [35]. This study has shown that ATE-EA was found to contain the flavonoids liquiritigenin, chrysoeriol, quercetin, naringenin, and quercetin-3,5,7,3’,4-pentamethylether. These flavonoids exerted an antiproliferative effect on human endometrial cancer cells along with the ATE-EA extracts.

Steroids are generally found in higher plants and have been used extensively against various diseases, including cancer [36]. The basic structure of steroids contains cyclopentanoperhydrophenanthrene with many substituents, including a hydroxy group at the C3 position, methyl at the C10 and C13 positions, and different side chains at the C17 position [37,38]. Based on the substituents at the C17 position, steroids are classified into cardiac glycosides, steroid saponins, phytosterols, and others [38]. The major dietary phytosterols are β-sitosterol, campesterol, and stigmasterol, with higher contents found in edible oils, seeds, and nuts. In this study, ATE-EA was found to contain the phytosterols β-sitosterol, campesterol, and stigmasterol. Epidemiologic and experimental studies also suggested the role of dietary phytosterols in protection from cancers, such as colon, breast, and prostate cancers [39]. In this study, our results demonstrate that the human endometrial cancer cells (HEC-1-A and RL95-2) were affected by the ATE-EA extracts as well as the mixed steroids. 

Adlay bran oil extracted by ethanol–water probably had higher proportions of palmitic acid (C16:0) and linoleic acid (C18:2) to oleic acid (C18:1) as well as potential higher anticancer function [40]. In addition, Xi et al. (2016) reported that the higher are the proportions of palmitic acid and linoleic acid to oleic acid ((C16:0 + C18:2)/C18:1), the higher are the growth inhibition rates on the cancer cell [41]. Our findings in the present study regarding major fatty acid contents of ATE-EA are consistent with the aforementioned studies.

## 4. Materials and Methods

### 4.1. Reagents and Materials

McCoy’s 5A medium, sodium bicarbonate, and dimethyl sulfoxide (DMSO) were purchased from Sigma-Aldrich (St. Louis, MO, USA). Paclitaxel, one of the most effective and widely used anti-cancer drugs for treating various tumors, was purchased from LC Laboratories (MO, USA). Bovine serum albumin (BSA), sodium bicarbonate, and 3-(4,5-dimethylthiazol-2-yl)-2,5-diphenyltetrazolium bromide (MTT) and other chemicals such as rhodamine-123, propidium iodide (PI), RNase A, sodium citrate, and standards for analysis were obtained from Sigma-Aldrich (St. Louis, MO, USA). All other chemicals and reagents for cell culture (DMEM/F12 medium, fetal bovine serum (FBS), epidermal growth factor, fibroblast growth factor, insulin, penicillin, and streptomycin) were purchased from Gibco (Grand Island, NY, USA). Coomassie Brilliant Blue R-250 was purchased from Bio-Rad (Hercules, CA, USA). Analytical-grade solvents used during the purification procedures, including acetonitrile, *n*-butanol (Bu), ethanol, ethyl acetate (EtOAc), hexane, and methanol were obtained from Merck (Darmstadt, Germany).

### 4.2. Plant Materials and Sample Preparation

The powder from all ground components was sieved through a 20-mesh sieve (aperture, 0.94 mm). The powder samples (100.0 g) were extracted with 10 volumes (*w*/*v*) of 95% ethanol (1.0 L), and filtration was used to separate samples at room temperature for 24 h. Then, dried ethanol extracts were obtained from the filtrate by concentration and stored at −20 °C. Ethanol extracts of four seed parts, namly the hull, testa, bran, and polish adlay of the adla seed, are referred to as AHE (ethanolic extracts of adlay hull), ATE (ethanolic extracts of adlay testa), ABE (ethanolic extracts of adlay bran), and PAE (ethanolic extracts of polished adlay), respectively.

### 4.3. Preparation of Ethanolic Extracts and Various Fractions from Adlay Testa

**Figure 8:**
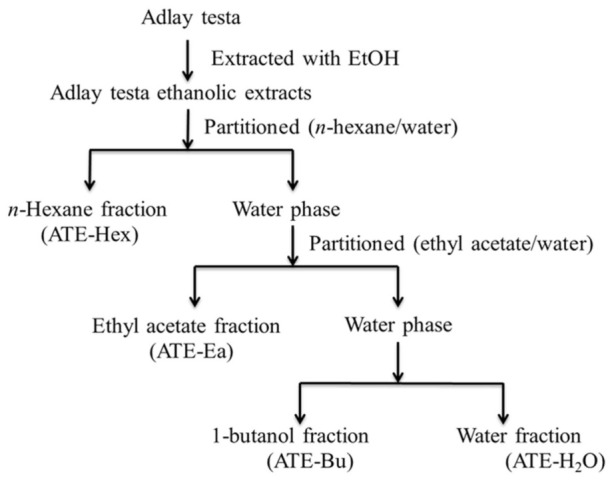
Extraction and partition scheme of adlay testa ethanolic extract (ATE).

As illustrated in Figure 8, the ethanolic extracts of the adlay testa were extracted and partitioned. Adlay testa powder (2.0 kg) was extracted with ethanol three consecutive times for 24 h and filtered. The filtrate, ethanol, and residues were separated in each 24-h extraction and concentrated to obtain 107.6 g (4.48%, based on the dry weight) of ethanolic extract under vacuum conditions. ATE, the ethanolic extracts of adlay testa, were suspended in water with 10% methanol and partitioned with hexane until the hexane fraction was colorless. Then, ATE-Hex (28.8 g, 1.2%) and ATE-EA (32.0 g, 1.33%) were obtained from the hexane fraction and dried under a vacuum or defatted by partitioning with ethyl acetate (EtOAc), respectively. Finally, ATE-Bu (10.0 g, 0.42%) from the residue in the butanoic fraction was obtained by partitioning with butanol and dried. ATE-Wa (24.4 g, 1.02%) was freeze-dried from the aliquoted fraction. Separation was done using silica gel and the mechanisms of isolation was based on the polarity of compounds. The different fractions were obtained from the EtOAc fraction by chromatographed successively on silica gel using eluting solutions with increasing polarity. ATE-EtOAc was subjected to column chromatography using silica gel and eluted with a Hex/EtOAc/MeOH gradient. Eight subfractions were obtained in the below combinations according to the similarity of each collection in thin layer chromatography (TLC): A (∼0–10% EA/Hex), B (∼10–20% EA/Hex), C (∼20–30% EA/hex), D (∼30–40% EA/Hex), E (∼40–50% EA/Hex), F (∼50–80% EA/Hex), G (∼80–100% EA/Hex), H (∼0–20% MeOH/EA), and I (∼20–100% MeOH/EA). The screening of the effective fractions was done through bioassay-guided fractionation procedures. 

### 4.4. Cell Lines and Culture

Human endometrial cancer cell lines (HEC-1A and RL95-2) were obtained from the Food Industry Research and Development Institute (FIRDI, Taiwan, ROC) and the Culture Collection and Research Center (CCRC, Taiwan, ROC). The HEC-1A cell line and RL95-2 cell line were cultured in McCoy’s 5A and DMEM/Ham’s F-12, respectively. All cells were maintained under the recommended conditions with 10% FBS and 1% antibiotics (10,000 units/mL penicillin, 10,000 μg/mL streptomycin, and 25 μg/mL amphotericin with 8.5 g/L NaCl). The atmosphere was 5% CO_2_ at 37 °C.

### 4.5. Cell Proliferation Analysis

The MTT assay was used to detect the cell proliferation effects of extracts of adlay seeds or paclitaxel. MTT was added to each well after the cancer cells were incubated in the presence of the extracts and paclitaxel treatment. The cancer cells (HEC-1A and RL95-2 cells) were at a density of 2000 cells/well with 100 μL of complete medium/well prepared before the above processing. An aliquot of 100 μL DMSO was added, and the plates were shaken and measured until the crystals dissolved at which point the absorbance was read at 570 nm on an ELISA plate reader (Model 550, Bio-Rad Laboratories, Hercules, CA, USA). The procedures of the MTT assay were performed as previously described [17,42]. The relative cell viability data were reported as the percentage of cells treated with the vehicle in at least triplicate experiments.

### 4.6. Cell Cycle Analysis

The easiest and rapidest method for detecting apoptosis is DNA staining with a propidium iodide (PI) solution and measurement in CellQuest Software with a FACSVantage (Becton Dickinson, Franklin Lakes, NJ) flow cytometry system. Cells treated with extracts from adlay seeds or paclitaxel were fixed in 75% ethanol at −20 °C. Cells were resuspended in 100 μL of phosphate buffered saline (PBS) containing 10 μL of RNase A (10 mg/mL) and incubated for 1 h at 37 °C after washing twice with cold PBS. The cells were then stained with 400 μL of PI (50 mg/mL) for 30 min at room temperature in the dark. For details of the procedures of cell cycle analysis by flow cytometry, see the work in [27,43].

### 4.7. HPLC Analysis

HPLC analysis was conducted on a Hitachi liquid chromatograph equipped with a L-6200 intelligent pump (Manasquan, NJ, USA) and L-7455 photodiode array detector (Manasquan, NJ, USA). Gradient elution was performed by varying the proportion of solvent A (water–acetic acid, 95:5 *v*/*v*) to solvent B (acetonitrile–acetic acid, 99.5:0.5 *v*/*v*), with a flow rate of 1 mL/min. A C18 guard column and a C18 reversed-phase packing column (250 × 4.6 mm 5 μM) were used (YMC Co., INC). Solvent B started at 10% for 10 min, then increased from 10% to 15% from 10 to 20 min, 15% to 16% from 20 to 35 min, 16% to 17% from 35 to 50 min, and finally 17% to 21% from 50 to 55 min. Phenolic compounds in the ATE-EA were identified based on the comparison of retention times and absorbance spectra at 280 nm to the reference standards using calibration curves.

### 4.8. GC Analysis 

GC/FID with a Varian 3400 GC (Palo Alto, CA) equipped with an EQUITY-5 column (30 m × 0.53 mm i.d., 0.15 μm film thickness) (Supelco, Saint Louis, MO, USA) was used for the composition analysis of steroids in the ATE-EA. Injector and FID temperatures were 320 and 330 °C, respectively. The carrier gas and makeup gas were hydrogens at a constant flow rate of 30.0 mL/min and nitrogen at flow rate of 28.0 mL/min, respectively. The temperature gradient had an initial temperature of 100 °C, then increased to 250 °C/min, held for 5 min, and then held at 300 °C for 30 min. 

We prepared a standard stock solution in hexane that included campesterol, stigmasterol, β-sitosterol, and stigmastanol. Different concentrations of work solutions were prepared daily by dilution a standard stock and stored at 4 °C. A solution of 0.05 mL cholesterol (2.0 mg/mL) as the internal standard (IS) was added to the EA fraction of the ATE (250.0 mg) in 5.0 mL hexane.

The fatty acid content was detected by promulgation for confirmation of national standards [44]. As shown in Table S1, palmitic acid, stearic acid, oleic acid, and linoleic acid account for the major content of ATE-EA.

### 4.9. Statistical Analysis

Data are presented as mean ± standard deviation. Determination of statistical significance was performed by one-way analysis of variance (ANOVA) and Dunnett’s multiple-comparison test using SPSS (IBM Corporation, Armonk, NY, U.S.A). Differences with *p* < 0.05 were considered significant. Results are representative of three independent experiments.

## 5. Conclusions

The results in this study show that adlay testa extract from adlay seed demonstrated remarkable inhibition of human endometrial cancer cell (HEC-1-A and RL95-2) growth. Moreover, ATE-EA induced G2/M arrest and sub-G1 phase accumulation. A cell proliferation assay using HEC-1-A and RL95-2 cells was employed to characterize the active compounds with potential antiproliferative effects of the ATE-EA. Furthermore, HPLC and GC/FID results reveal potent antiproliferative effects of phenolic compounds, flavonoids, steroids, and fatty acids on human endometrial cells. These identified compounds should be considered new natural resources with potential therapeutic benefits against endometrial cancer. Taken together, the ATE-EA studied in this work can be served in functional foods and health products with potential benefits against endometrial cancer.

## Figures and Tables

**Table 1:** Detection and quantification of the phenolic compounds from ATE-EA by HPLC.

Compound (μg/g)	ATE-EA
280 nm	
Protocatechnic acid	557.0572
*p*-Hydroxybenzoic acid	299.6813
Chlorogenic acid	6293.503
Vanillic acid	1836.49
*p*-Hydroxybenzaldehyde	213.8971
Syringic acid	585.5772
Vanillin	782.389
Syringaldehyde	1500.096
320 nm	
Caffeic acid	239.356
*p*-Coumaric acid	N.D.
Ferulic acid	N.D.

N.D. = not detected.

**Table 2:** Detection and quantification of the flavonoid compounds from ATE-EA by HPLC.

Compound (μg/g)	ATE-EA
Homoeriodictyol	N.D.
5,7-dihydroxy chromone	N.D.
Formononetin	N.D.
Kaempferol	N.D.
Nobiletin	N.D.
Isoliquiritigenin	N.D.
Tangeretin	N.D.
Liquiritigenin	7624.774
Quercetin	145.785
Naringenin	1058.989
Chrysoeriol	159.576
Quercetin-3,5,7,3’,4-pentamethylether	48.793

N.D. = not detected.

**Table 3:** The concentration of phytosterols in ATE-EA.

Phytosterols	Molecular Weight	Retention Time (min)	Relative Retention Time ^1^	Peak Area	Peak Area Ratio ^2^	RRF	Conc (μg/g)
Cholesterol	386	18.023	1.0000	28263			
Campesterol	400	19.813	1.0993	14342	0.5074	0.89	**1112.38**
Stigmasterol	412	20.375	1.1305	13385	0.4736	0.81	**1163.13**
Β-sitosterol	414	21.498	1.1928	70528	2.4954	0.87	**5696.74**
Stigmastanol	416	21.737	1.2061	17810	0.6302	0.78	**1711.64**

^1^ Retention times relative to cholesterol. Cholesterol was used as the internal standard. ^2^ The concentration of the phytosterol standard mixture solution was 1000 µg mL^−1^.

## Data Availability

Data are contained within the article and the Supplementary Materials.

## Supplementary Materials

The following are available. Figure S1. Chromatogram of a phenolic compounds (A) and ethyl acetate fraction of the adlay extract (ATE-EA) monitored at 280 nm.1: protocatechnic acid. 2: ρ-hydroxybenzoic acid. 3: chlorogenic acid. 4: vanillic acid. 5: ρ-hydroxybenzaldehyde. 6: syringic acid. 7: vanillin. 8: syringaldehyde. 9: caffeic acid. 10: *p*-coumaric acid. 11: ferulic acid. Figure S2. Chromatogram of a phenolic compounds (A) and ethyl acetate fraction of the adlay extract (ATE-EA) monitored at 320 nm. 1: protocatechnic acid. 2: ρ-hydroxybenzoic acid. 3: chlorogenic acid. 4: vanillic acid. 5: ρ-hydroxybenzaldehyde. 6: syringic acid. 7: vanillin. 8: syringaldehyde. 9: caffeic acid. 10: *p*-coumaric acid. 11: ferulic acid. Figure S3. Peaks were identified from the retention times of standard compounds (A) and ethyl acetate fraction of the adlay extract (ATE-EA) (B). 1, cholesterol; 2, campesterol; 3, stigmasterol; 4, β-sitosterol; 5, stigmastanol. Cholesterol was used as the internal standard. Table S1. Detection and quantification for the contents of fatty acid compounds from ATE-EA. Table S2. The retention time, regression equation and regression coefficient of the phenolic compounds. Table S3. The retention time, regression equation and regression coefficient of the flavonoid compounds.

## Sample Availability

Samples of the crude extract prepared from *Coix* seed are available from the auothors.
