# Supplementary material for: Adlay Testa (Coix lachryma-jobi L. var. Ma-yuen Stapf.) Ethanolic Extract and Its Active Components Exert Anti-Proliferative Effects on Endometrial Cancer Cells via Cell Cycle Arrest
Source: Molecules. 2021 Mar 31;26(7):1966. doi: 10.3390/molecules26071966 (PMC8037553; doi:10.3390/molecules26071966)
Supplement: Supplementary file 1 [file molecules-26-01966-s001.pdf]

Supplementary Materials:

(A)

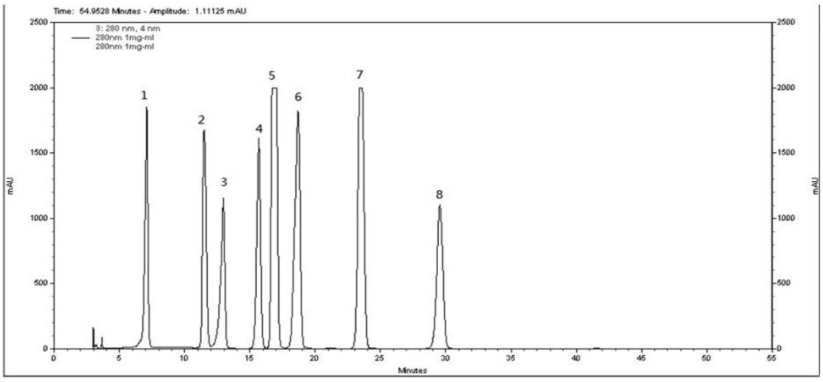

(B)

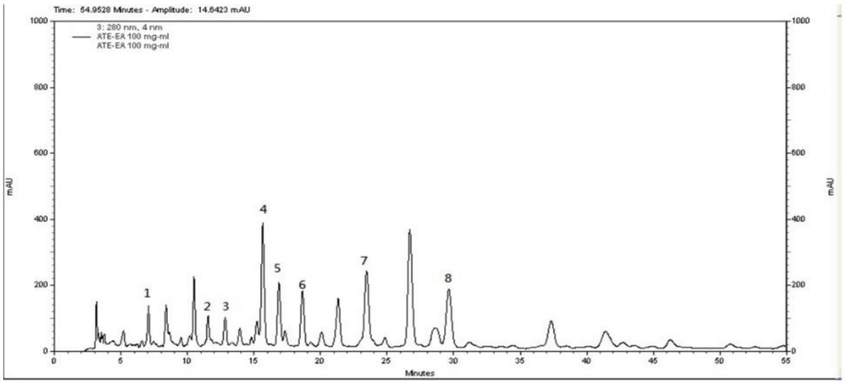

Figure S1. Chromatogram of a phenolic compounds (A) and ethyl acetate fraction of the adlay extract (ATE-EA) monitored at 280 nm.1: protocatechnic acid. 2: ϱ-hydroxybenzoic acid. 3: chlorogenic acid. 4: vanillic acid. 5: ϱ-hydroxybenzaldehyde. 6: syringic acid. 7: vanillin. 8: syringaldehyde. 9: caffeic acid. 10: p-coumaric acid. 11: ferulic acid.

(A)

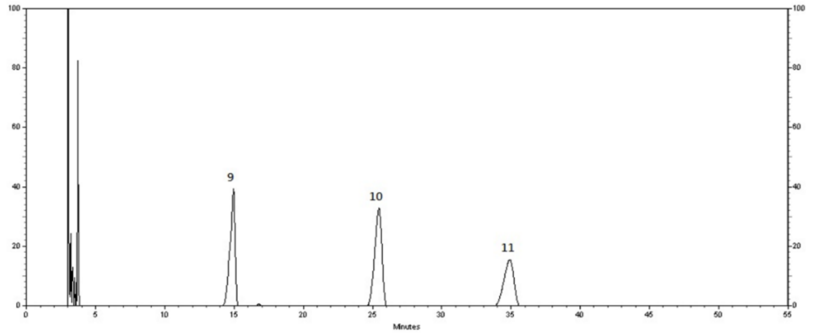

(B)

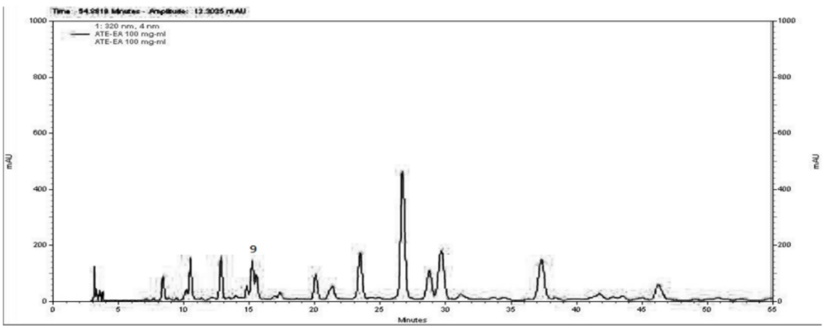

Figure S2. Chromatogram of a phenolic compounds (A) and ethyl acetate fraction of the adlay extract (ATE-EA) monitored at 320 nm. 1: protocatechnic acid. 2: ϱ-hydroxybenzoic acid. 3: chlorogenic acid. 4: vanillic acid. 5: ϱ-hydroxybenzaldehyde. 6: syringic acid. 7: vanillin. 8: syringaldehyde. 9: caffeic acid. 10: p-coumaric acid. 11: ferulic acid.

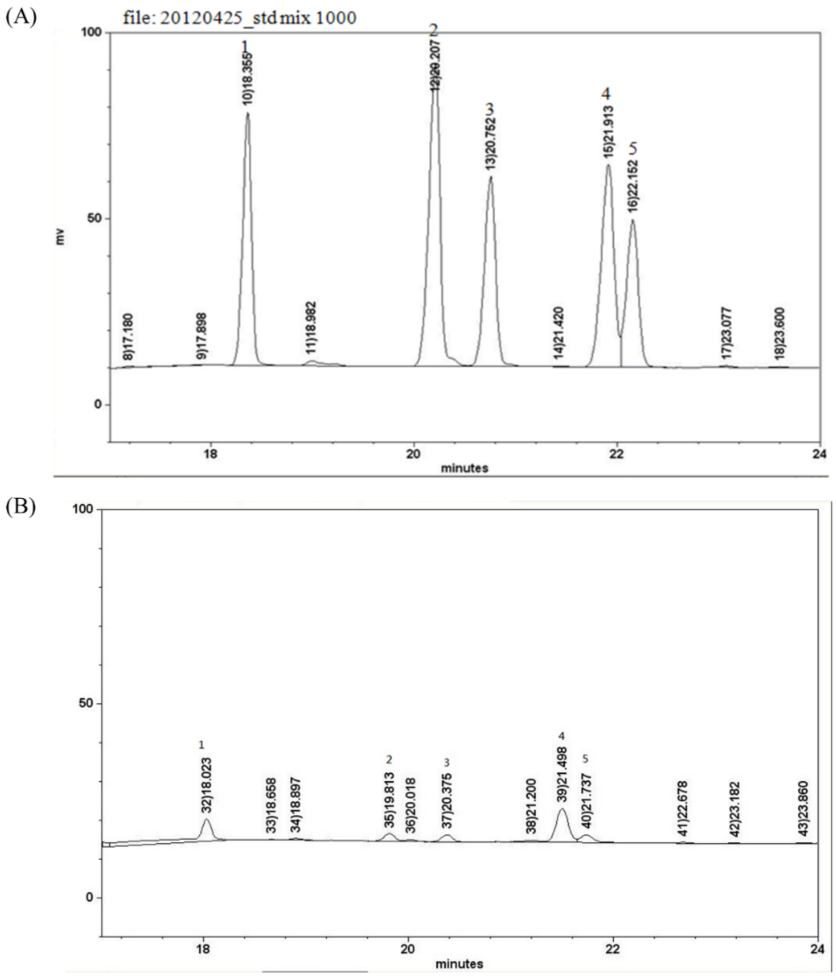

Figure S3. Peaks were identified from the retention times of standard compounds (A) and ethyl acetate fraction of the adlay extract (ATE-EA) (B). 1, cholesterol; 2, campesterol; 3, stigmasterol; 4, β-sitosterol; 5, stigmastanol. Cholesterol was used as the internal standard.

Table S1. Detection and quantification for the contents of fatty acid compounds from ATE-EA.

Compound (mg/g)	ATE-EA
8:0 Caprylic acid	N.D.
10:0 Capric acid	N.D.
12:0 Lauric acid	N.D.
14:0 Myristic acid	N.D.
14:1 Myristoleic acid	N.D.
16:0 Palmitic acid	161
16:1 Palmtoleic acid	N.D.
18:0 Steari acid	12
18:1 Oleic acid	467
18:2 Linoleic acid	345
18:3γ-linolenic acid	N.D.
18:3α- linolenic acid	N.D.
20:0 Arachidic acid	N.D.
20:1 Eicosaenoic acid	N.D.
20:2 Eicosadienoic acid	N.D.
20:3γ-Eicosatrienoic acid	N.D.
20:3α-Eicosatrienoic acid	N.D.
20:4 Arachidonic acid	N.D.
20:5 Eicosapentaenoic acid	N.D.
22:0 Behenic acid	N.D.
22:1 Erucic acid	N.D.
22:2 Docosadienoic acid	N.D.
22:4 Docosatetraenoic acid	N.D.
22:5 Docosapentaenoic acid	N.D.
22:6 Docosahexaenoic acid	N.D.
24:0 Lignoceric acid	N.D.
24:1 Nervonic acid	N.D.
Others	14

N.D.: Not detected.

Table S2. The retention time, regression equation and regression coefficient of the phenolic compounds.

Compound	Retention time (min)	Regression equation	Regression coefficient (r^2)
280nm			
protocatechnic acid	7.02	y=108568732.9x+1181073.69	0.9999
p -hydroxybenzoic acid	11.54	y=129553867.1x+2594215.43	0.9981
chlorogenic acid	12.813	y=103073849.9x+355106.94	0.9999
vanillic acid	15.653	y=136177380.9x+929389.7	0.9997
p -hydroxybenzaldehyde	16.86	y=618704222.2x+582145.44	0.9999
syringic acid	18.627	y=199333462.7x+1678917.23	0.9996
vanillin	23.46	y=339212539x+305480.72	0.9999
syringaldehyde	29.627	y=140613999.3x+769250.19	0.9999
320nm			
caffeic acid	15.173	y=309350875x+5427362.73	0.9957
p -coumaric acid	25.893	y=382978154x+7472195.23	0.9969
ferulic acid	34.373	y=373821554.3x+1737533.3	0.9997

Table S3. The retention time, regression equation and regression coefficient of the flavonoid compounds.

Compound	Retention time (min)	Regression equation	Regression coefficient (r^2)
Liquiritigenin	7.1	y=1615.17x+32171.73	0.9856
Quercetin	8.14	y=89295.84x-1214520.38	0.9879
Naringenin	10.913	y=10218.08x-25052.23	0.9976
Chrysoeriol	12.207	y=67636.98x+31111.93	0.9994
Quercetin-3,5,7,3',4-pentamethylether	20.307	y=124605.57x-465334.24	0.9891
